# JU:MP leads: sparking physical activity leadership and supporting positive youth development in a deprived community

**DOI:** 10.3389/fspor.2024.1490688

**Published:** 2024-12-19

**Authors:** Jamie Crowther, Elliot Lever, Sufyan Dogra, Sally Barber, Jennifer Hall

**Affiliations:** Bradford Institute for Health Research, Bradford Teaching Hospitals NHS Foundation Trust, Bradford Royal Infirmary, Bradford, United Kingdom

**Keywords:** young leaders, physical activity, positive youth development, deprived community, community

## Abstract

**Introduction:**

Physical activity interventions in deprived communities should acknowledge the social, political, and cultural context in which they are delivered. Targeted young leaders programs can harness positive youth development principles and address these concerns by engaging underrepresented young people and developing them as physical activity leaders who can support local delivery efforts. Community-based Youth Leadership development programs are under-researched, and little is known about how to develop young people from deprived communities as physical activity leaders.

**Methods:**

This research project used interviews, focus groups and Ripple Effects Mapping to evaluate a community based young physical activity leaders development program delivered in a community with high levels of deprivation in Bradford, UK.

**Results:**

The program, known as “JU:MP leads” developed 20 young people aged 16-25 as young leaders between September 2022 and September 2023. Thematic analysis of data uncovered that a community-based young leaders development program can be effective in supporting local young people from a deprived community to develop as physical activity leaders, particularly when adopting a flexible delivery model through which young people can engage around other commitments. Key mechanisms within the program that supported development included the acquisition of formal, nationally recognised qualifications, informal training and mentorship, peer support and ongoing reflection. These key mechanisms of the program facilitated the personal and professional development of the young people into confident and assured physical activity leaders.

**Discussion:**

The research concluded that community based physical activity leaders programs can support Positive Youth Development of young people from deprived communities by developing their skills and supporting them to make valid contributions to local physical activity provision.

## Introduction

Physical activity is vitalfora healthy lifestyle and is essential for young people's health and development. Physical activity contributes to many markers of youth health including cardiovascular function, psychological well-being and healthy weight (1). Yet, many young people in deprived communities in the UK (2) and globally (3, 4) don't meet physical activity guidelines. Interventions in these communities are most effective when they consider the local social, political, and cultural contexts (5). Relatability between coaches and participants is identified as a mechanism for supporting physical activity engagement (6–8). One method to achieve this is to develop local young people as physical activity leaders (9). Such an approach is considered to have notable potential to support local physical activity efforts, particularly as it has been highlighted that the success of such efforts in deprived communities often does not lie in the activities being delivered, but more in the social capital that is mobilised and formulated by them through the development of interpersonal connections (10). This paper evaluates a program seeking to develop young leaders and therefore beyond acknowledging this, the broader social capital approach provides limited utility as it focuses on its functionalist role in promoting inclusion and development. Instead, a Positive Youth Development perspective is favoured to capture the nuanced developmental nature of the program. The term deprived community used in this article is used to refer to a community that experiences limited access to essential resources, has historically had poor infrastructure relating to physical activity and health, and where social mobility has subsequently been limited (11).

In the literature, terms including leader, coach and mentor are highlighted as often being used interchangeably regarding physical activity leadership (12, 13). In this paper, “young leader” describes a young person who is engaged in coaching, organising, and facilitating physical activity or is pursuing physical activity leadership in the future. This distinction aligns youth physical activity leadership with PYD and social justice,framing leadership as an attainable goal for all young people to contribute to now and in the future (14, 15).

### The potential of young physical activity leader’ programs

Community-based young physical activity leadership development is under-researched, with limited understanding of its impact mechanisms (16). Additionally, women are underrepresented in physical activity leadership roles, as such roles compete with societal gender norms that position women as primary caregivers (17, 18). Thecentrality of adult leaders under-acknowledges the importance of relatedness in the youth physical activity context. Research has shown that leader-relatedness facilitates feelings of safety, in turn, supporting young people's physical activity engagement and satisfaction (19, 20). One solution to these issues isdeveloping young people as physical activity leaders from within communities (21). Community settings can harness the potential of different markers of cultural identity to support physical activity programs (22). Targeted Young Physical Activity Leaders’ Programs (herein called Young Leaders programs) can support the engagement of underrepresented groups of young people in physical activity leadership and may more broadly support increased physical activity opportunities in deprived communities (23, 24). However, there is an inherent tension in delivering such interventions in deprived communities as it isevidenced that such commcussionunities face multifaceted and interrelated barriers when accessing physical activity (25). Therefore, such programs may be limited in their impact and possibly detrimental to the young people seeking to lead physical activity unless they are matched with wider systemic support. This was not a prominent issue with JU:MP leads as the program was delivered as part of a whole-systems approach to increasing physical activity (26). Therefore, it was acknowledged that the community was resourced to facilitate the program.

Young people are underutilised community assets that have scarcely been harnessed for promoting individual, local and societal level change (27). Aligned with Positive Youth Development (PYD), Young Leaders programs can harness young people's inherent motivation, energy and enthusiasm towards supporting local physical activity (14, 28, 29). PYD sees young people as possessing strengths that can be harnessed for social good and as such aligned leadership programs are about harnessing young people's capacity for leadership in the present (30). PYD philosophy recognises that the development of the 5Cs (Competence, confidence, connection, character, caring) motivates the 6th C, Contribution, to self and community (31). Aligned with PYD and social learning theory (32), developing young people as physical activity leaders serves a dual benefit in supporting young people's social progression through joint endeavour whilst facilitating opportunities to serve the community and provide inspiration for peers and local young people through leadership (14, 33–35). Youth are acknowledged as facing social justice-based adversities such as limited opportunities for social progression (36, 37). PYD and Youth Leadership based approaches can be effective means of supporting young people in tackling these challenges head on (30, 38). Yet more research is needed to understand how such approaches can work with different youth populations such as those from ethnic minority groups.

### Young physical activity leader programs: distinctions and approaches

Within the physical activity literature, most exploration of Youth Leadership focuses on the process of engaging young people as volunteers within the physical activity workforce (39, 40). This study shifts the focus to a program where young people were employed (paid), aligning with recent calls for community-based leadership opportunities (40). This is significant because volunteer focused Young Leaders initiatives often contribute to idealised misrepresentations of activism (41) and such approaches are inappropriate within the context of a deprived community as they fail to subvert the structural contributors of deprivation (42). Qualitative studies advocate investing in young people, staff, volunteers, and grassroots community groups who are direct and first-hand beneficiaries of targeted community-based interventions to reduce inequalities, including in physical activity (43). This process supports an ideological shift in youth-led community-based physical activity delivery, away from social activism and towards market-driven delivery (40). Employment opportunities can professionalize the youth leader workforce, help youth enter the physical activity sector, and position leadership as a viable career (42).

Youth sport and physical activity leadership programs vary in delivery approaches (44). The most prominent in the UK are those delivered by the Leadership Skills Foundation [formerly Sports Leaders UK (SLUK)], who provide voluntary leadership opportunities, in which young people can gain nationally recognised qualifications (39). SLUK, similar to the majority of UK providers, delivers a structured program centred on formal education, experiential learning, and supervised leadership. Mawson et al. (39) have been critical of SLUK for its overreliance on delivering in educational establishments. They recommend greater efforts be made to engage young people beyond education contexts, arguing communities will benefit from an embedded physical activity leaders workforce (39). School-based approaches often fall within the *youth services approach—youth leadership* categories of the youth engagement continuum (45) in the sense that they define young people as clients, are designed to meet specific outcomes, usually, increasing individual engagement in physical activity and feature basic components of youth leadership. However, they rarely aim for braoder systemic change

### Community-based young physical activity leader development programs: advantages, challenges, and knowledge gaps

We identified three community-level young physical activity leader interventions within the literature; two for low-income youth in North America and one for young females in Scotland (14, 46, 73). These studies evidence the feasibility and benefits of community-based young physical activity leader development butdid not explicitly engage young people experiencing multiple layers of disadvantage (47). A recent Delphi study to develop consensus statements for young physical activity leader development reported that community-based programs have potential to recruit underserved youth, and are therefore better placed to address local issues (13). Flexibility and diversity in these programs help create real-world opportunities (13). Community-based programs are also better placed to engage underrepresented groups of young people (39). Thus by supporting representation such programs can challenge the stratification of the overall physical activity workforce including, coaches, organisers and managers which, like the broader physical activity landscape, has historically been built on unequal representation in relation to several characteristics including socio-economic status, race and gender (4, 39). Moreover, such programs, particularly where focused on engaging underserved populations are well placed to develop young people towards forms of collective empowerment and systemic change (45, 48). Steward et al. (13) noted that in the community context, programs should be delivered flexibly to increase their relevance and accessibility to the young people they seek to serve, and be set up to account for the complexity and diversity of community contexts. Despite this recent investigation, community-based Youth Leadership development is under-researched, and little is known about the mechanisms of impact involved in such programs (16).

## Methodology

This paper meets the call for understanding of community based Youth Leadership development programs by sharing findings from an empirical inquiry into the JU:MP leads program. The program was a community-based young physical activity leaders program delivered in Bradford, a deprived and multi-ethnic city in the North of England. The research described within this paper supported evaluation of the JU:MP leads program from the experience of youth participants and program staff. The research aimed to:

(1) Examine how a physical activity leadership development program, delivered in a deprived community can reduce inequalities in accessing physical activity leadership development for young people aged 16–25.
(2) Investigate program effectiveness in empowering local youth to become physical activity leaders and identify key mechanisms contributing to leadership development.
(3) Examine the factors that influenced program implementation.

### Study design

This study employed a multi-method qualitative case study design to explore the JU:MP leads program. Multi-method qualitative design is well established in the field of physical activity. By integrating multiple qualitative methods, including in-depth interviews, focus groups, and Ripple Effects Mapping (Herein REM), we holistically explored physical activity leadership development within the JU:MP leads program (49). This approach allowed for an in-depth examination of the mechanisms and outcomes from the program and enabled the identification of themes that may not have been evident through a single method (50). The use of multiple qualitative techniques supported methodological rigour and enhanced the credibility and robustness of the findings.

A realist evaluation approach was taken to facilitate the development of nuanced understandings of what worked, for whom, within the localised context (51, 52). The rationale for the realist approach was to explore the program from a position that facilitated understandings of how the program was operating locally within Bradford, and how different organisations and young people experienced the program (53). The realist perspective permitted exploration into the divergence between the context and practice of the varying LTO's.

### Study context: the JU:MP leads program

The JU:MP leads program (herein referenced “the program”), was a community-based Young Leaders program delivered as part of the Sport England-funded Bradford Local Delivery Pilot (LDP) known as Join Us, Move Play (JU:MP). Taking a systems-based approach, JU:MP seeks to increase physical activity within a neighbourhood in North Bradford, England (26). JU:MP commissioned the program as part of its systems-based approach to increasing physical activity. The program was delivered to 20 young people aged 16–25 from the LDP area with the rationale that representative, professionally trained Young Leaders could increase local physical activity opportunities and serve as role models for local children and young people. The program focused recruitment exclusively on members of the Black and Minority Ethnic (BAME) community with a particular focus on recruiting women and girls. The rational being to reduce inequalities in the field of physical activity leadership. The aim of the program was to develop dynamic and inspirational local leaders who can be positive peers and contribute to the development of sustainable physical activity, thus creating an inspirational legacy within the LDP area. The program was purely community-based, meaning unlike previously researched young physical activity leaders programs it was delivered with no affiliation to educational institutions which is an important distinction from previous research as it means all aspects of the program including recruitment and development took place within the community (39).

JU:MP commissioned national doorstep sport provider StreetGames, in partnership with four Local Trusted Organisations (LTOs), to manage the delivery of the project. StreetGames did this under their #NextGen development package. StreetGames (74) states that #NextGen works to equip young people from underserved communities with the tools to transform their futures, and the organisation's focus is to harness the power of sport and physical activity to improve the lives of children and young people living in the UK's most underserved communities. StreetGames allocated a program manager to the JU:MP leads program who held responsibility for managing progress across the four LTOs and providing a core training program of nationally recognised qualifications comprising Level 2 Multi Skills, Safeguarding, Young people's mental health awareness, and First aid to upskill aspiring Young Leaders.

**Figure 1:**
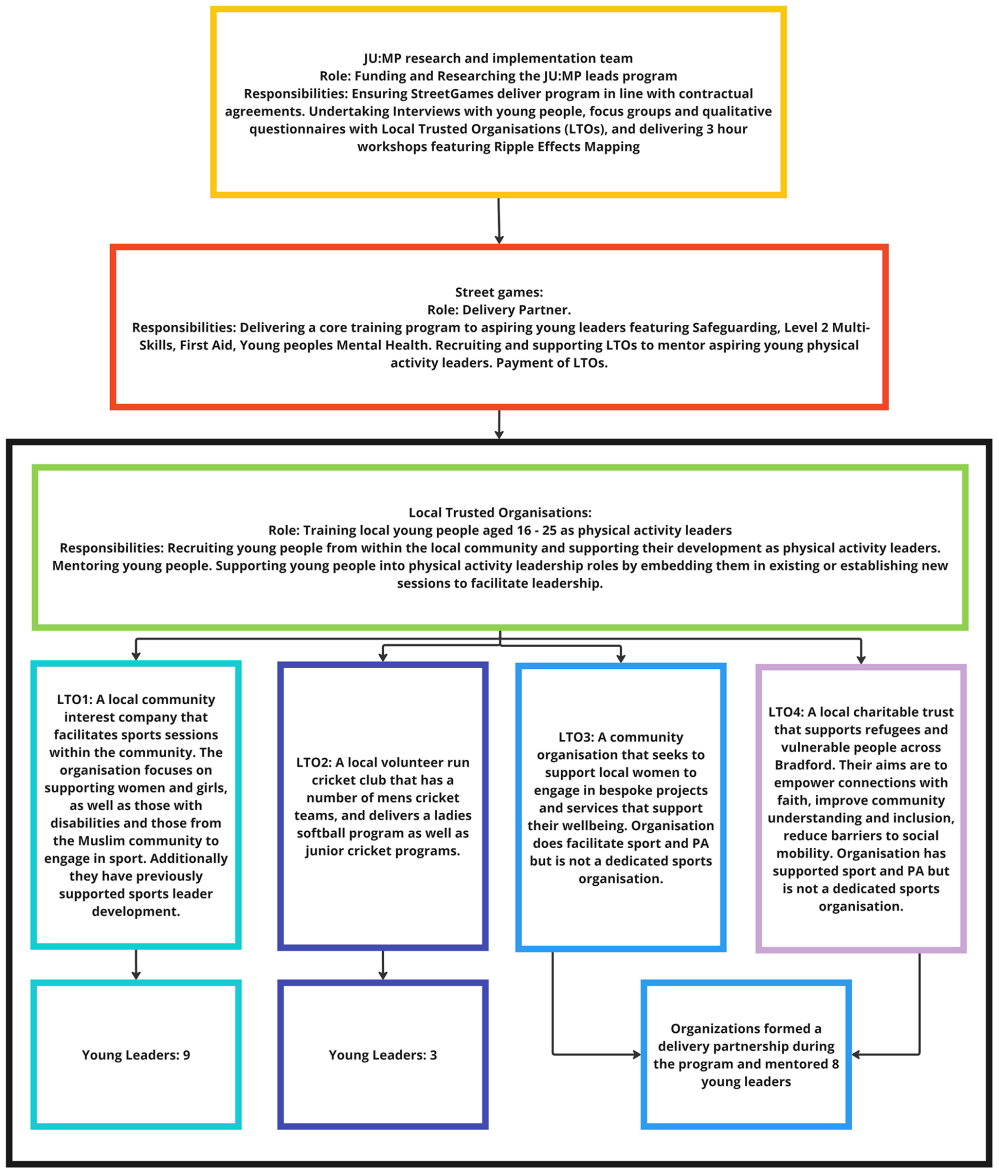
Program structure and roles and responsibilities.

StreetGames deemed that the four LTOs had the expertise to recruit and support a total of 20 young people in developing as physical activity leaders. This decision was based on the LTOs position and reach within the local community and on their track record of engaging young people in development programs. Two of the organisations were specialist physical activity organisations meaning their core function was to deliver physical activity, and two were not. Aspiring young physical activity leaders were recruited to the program by their respective LTO based on them having the motivation and desire to develop as physical activity leaders. LTOs were contractually obliged to provide mentorship for the Young Leaders and provide them with six hours of physical activity delivery for at least 30 weeks. 20 young people ranging from ages 16–25 were recruited by LTOs to participate in the program; eight were females and 12 were male. All young people were South Asian, andwere engaged in either full-time education or full/part-time employment. 12 young people were mentored by the physical activity organisations and eight young people were mentored by community organisations without a physical activity specialism. Figure 1 provides a detailed overview of the program structure.

### Ethics

Data was gathered over 12 months between September 2022–2023. Ethical approval was obtained from the University of Bradford on 20/04/21, amendments were approved 20/11/22 (**Ref:E877**). Owing to the methodology of the research, in particular the collaborative and open nature REM, internal confidentiality and anonymity was a concern. However, as REM focused on collectively mapping tangible events and their interconnections with no sensitive topics being discussed, it was felt that breaches of anonymity and confidentiality were of minimal risk. Concerning the anonymity of interview participants, a unique participant ID was used for data storage, with pseudonyms being used in this paper to protect the participants’ identities.

### Consent

Consent for research was obtained from all participants by JC who explained to each participant their role in the research and permitted opportunities to ask questions. All LTOs were contacted directly by JC. Access to young people was facilitated by mentors from LTOs who acted as gatekeepers (54).

### Sampling

Purposive sampling was used to recruit participants for focus groups and interviews (55, 72). All young people and deliverers that were involved in JU:MP leads were invited to participate in this research. All mentors from LTOs (*n* = 4) and a StreetGames representative were invited to participate in focus groups based on their experience delivering the JU:MP leads program and in mentoring aspiring Young Leaders. A subsample of young people (50%, *n* = 10) were selected for interview. Young people were identified following a request from the lead researcher (JC) to the LTOs to identify young people for interview. The request featured guidance to include a spread of young people based on gender, age, perceived competence and life circumstances (in education, other employment). The details of Young Leaders identified for interviews were passed to JC who contacted each individually to make arrangements for interviews. It was possible that our request to LTO's may have resulted in some bias in the sample as LTOs may have selected young people who they felt would speak favourably of their engagement in the program. However, as all but one young person engaged in the program for the duration of the delivery period, we believed all such young people were ’success stories’ and would therefore provide a balanced account of their experience. The young person who dropped out of the program did so between their first and second interview. We still managed to engage them in a follow up interview via WhatsApp, meaning we were able to capture the experiences of the only participant who withdrew from the project.

### Data collection

Table 1 provides an overview of which data collection methods were engaged in by which participants and at which time point within the research.

**Table 1:** Research participants and methods.

		Participants		
		Young leaders (*n* = 20)	Mentors (*n* = 4)	Street games representative (*n* = 1)
Data collection method	Ripple Effects Mapping	All 20	All 4	—
	One-to-One Interview	Subsample (50%)	—	—
	One-to-One Interview	Same Subsample (50%)	—	—
	Focus Groups 1. December 2022 2. March 2023 3. June 2023 4. September 2023	—	All 4	June 2023 and September 2023
	Qualitative Questionnaire	—	—	—

### Interviews

Semi-structured interviews were conducted with the young people based on realist principles, using an interview guide based on the RAMSES II guide to realist interviewing (56). Interviews lasted from 45 to 60 min. Interviews took place in community centres within the JU:MP area or online with the time, format and location being arranged based on the views of the participants. Interviews were planned for two time-points, halfway through and just before the end of the program. Our intention was for the same subsample of participants to take part in both interviews for consistency and to allow opportunity to explore change. During the first round of interviews questions were focused on exploring the how and why of engagement, and personal and organisational progress and development. During the second round of interviews, questions were more reflective and directed participants to reflect on their experiences within the program and outline any impacts, their significance and any contributing mechanisms.

JC conducted all 10 interviews in the first round of interviews (February 2023). JH conducted three, EY conducted four and EL and KF each conducted one interview in the second round (September 2023). One interview in the second round of interviews (September, 2023) was conducted via WhatsApp messages as the young person had dropped out of the program and was no longer available for a face-to-face interview.

### Quarterly evaluation and learning workshops

Four evaluation and learning workshops were delivered during the program delivery period (December, 2022, February, 2023, May, 2023, August, 2023). These workshops were scheduled on a weekday evening to ensure young people were able to attend in addition to their work or education commitments. Theyprovided a space for StreetGames, LTOs and young people to come together and share their experiences and learning and were conducted in community venues in line with the community based ethos of the program. Workshops lasted three hours and included time for steering group meetings to discuss progress with delivery including more practical elements of the program such as attendance at training and number of hours delivered by young leaders, focus groups to explore specific elements of the program in depth including challenges with facilitating physical activity sessions, social time with food provided, and REM. The workshop itinerary was as follows:

• 30 min for Steering Group meeting
• 45 min for Focus group with LTOs and StreetGames Representatives
• One hour for Introduction/recap of REM and delivery of REM session
• 45 min for Social time and food provided

These workshops were intended to integrate research with delivery and increase engagement in research. LTO mentors and a representative from StreetGames were present for the duration of the workshop, whereas young people were only in attendance for the social time with food, and REM.

### Focus groups

Four mentors from the Local Trusted Organisations engaged in a focus group at four time points in the program (December, March, June, September). The program manager allocated for the JU:MP leads program by StreetGames participated in the focus group at two time points (March and September). The focus group guide was based on realist principles and the RAMSES II guide (56). Focus Groups lasted from 45 to 60 min. Focus groups with LTOs took place at the beginning of the three-hour workshops. JC conducted the first three focus groups and JH conducted the last.

### Ripple effects mapping (REM)

REM was embedded into the quarterly workshops to map key events and impacts (75), to capture program mechanisms and explore the relationship between actions and impacts (57, 76). REM offered an opportunity to map progress within the program as well as an opportunity to map the wider impacts of the program and an opportunity to explore what events contributed to different outcomes (57). Up to one hour within each workshop was allocated to REM. The benefit of embedding REM alongside focus groups and steering group meetings was that prompts could be added to the Ripple Effects Map prior to young people arriving at the workshop, thus facilitating discussion.

In conducting REM, methods were adopted from published research (57). With the intention of capturing potential diversity of delivery within the project we made the decision to develop two Ripple Effects Maps. We split the group so that two LTOs and their young people would contribute to one map, and the other two would contribute to another. During the first workshop, participants were presented with a roll of paper and instructed to draw a line down the middle to represent the timeline of the project. Following this, participants were instructed to begin adding events such as training, delivery and recruitment for the project. Participants were given free reign to contribute anything they felt relevant to the map. Once events had been contributed, participants were encouraged to draw connections between them to indicate a possible contribution and attribution relationship. Questions such as “what impacts have happened as part of the program” and “what event helped contribute to that impact” supported this. The physical activity-based LTOs contributed to one map, whilst the non-physical activity-based LTOs contributed to another. This decision was based on the thinking that all LTOs had different “journeys” and we wanted to capture them somewhat independently but still allow enough space for critical discussion. At proceeding workshops the same Ripple effects maps were added in order to capture the entirety of the project.

### Qualitative questionnaire

All LTOs completed a qualitative questionnaire reflecting on their experiences and progress at quarterly intervals. LTOs were sent the qualitative questionnaire one week in advance of the evaluation workshops and asked to return them during the workshop. Questionnaires featured four prompts:

1. What progress have you made on delivering the program?
2. What impacts have you noticed?
3. What has influenced delivery and impact?
4. Learning and next steps.

### Data analysis

Reflexive thematic analysis was used to analyse the data gathered through the multi-method research approach (58). The process supported the identification of commonalities across the data. Analysis was engaged in recursively by authors JC and EL. The first step of familiarisation was achieved through JC and EL transcribing interviews and focus groups, inputting qualitative questionnaire responses into a electronic database on Excel, and synthesising the completed Ripple Effects Map into one electronic map via the online workspace MIRO. Further familiarisation occurred through JC and EL reading all transcripts and questionnaire responses in full and taking time to discuss the synthesised Ripple Effects Map. Once a good grasp of the data was achieved, JC and EL moved through the stages of coding, developing themes, and reviewing and defining themes iteratively, ensuring inter-rater reliability by researchers JC and EL both independently coding the data. To support consistent intercoder agreement, JC and EL met periodically to review their codes and themes and discuss and defend their thinking, only stopping once congruence had been achieved (59). Peer debriefing was engaged in (60) by draft themes being shared with JH for sense checking. This process supported the rigour of our analysis by providing opportunity to refine and confirm our interpretations of the data, and engage in critical reflexivity about our data analysis (61).

### Rigour

The academic rigour of this research was established through multiple processes. Firstly, the various data collection methods used within this research support the overall credibility and rigour of the study (62). This was through each method supporting a fuller understanding of the program with the diverse approaches to data collection providing an opportunity to explore gaps in understanding that were uncovered by the other means of inquiry. The research team consulted the wider Physical activity research group situated at Born In Bradford, and members of the Bradford Centre for Qualitative research to sense-check their research plans at the beginning of the research and findings at the end. This was done through a presentation of the research and its findings to each group. Overall, these groups helped ensure that research aligned with recommendations for rigorous qualitative research as outlined by Tracy (63) and Smith and McGannon (61) including exploring a relevant and timely topic by providing an in-depth evaluation of a real world project in a timely manner. Also, providing a detailed description of the project, research processes and findings, and being delivered with meaningful coherence.

### Positionality

The data collection team consisted of JC, EL, JH, EY who are all white British and live outside, but local to Bradford, and KF who is South Asian and lives in Bradford. These distinctions are important because the JU:MP leads were exclusively South Asian and living in Bradford. JC and EL are male, and JH, EY and KF are female. JC and JH were in their early 30s, and EL, EY and KF were within the age range of the Young Leaders (16–25) at the time of data collection. Where possible we attempted to match the sex of the researcher with the sex of the research participant, yet this was not always possible. Whilst the data analysis team, comprising JC, EL, JH have a solid understanding of the challenges faced by the South Asian community in accessing physical activity, this knowledge is developed from them being embedded in the wider JU:MP Local Deliver Pilot, and is not based on experiential knowledge. Thus, it is notable to acknowledge that the research team, with the exception of KF were outsiders in the research in that they were white British and had no lived experience of being a young person growing up in Bradford and engaging in physical activity in that context (64). This reality is important to acknowledge as it may have influenced the data that was collected, how it was analysed and how it is presented herein (65). Moreover, as the research team was mixed gender, this resulted in instances where male researchers who are White British, were interviewing female Young Leaders of south asian heritage, and female researchers who are White British were interviewing male Young Leaders of South Asian heritage. This in particular could have impacted the data gathered, particularly where data was collected by researchers around the intersectionality of ethnicity and sex.

## Findings

In total 20 young people aged 16–25 engaged in the program. Eight were female and 12 were male. All young people engaged in the program in addition to other work and/or education commitments. All young people were recruited by Local Trusted Organisations (LTOs) with support from StreetGames. Young people were either recruited through LTO existing local networks or through open calls for applications.

Overall there were four themes drawn out from the thematic analysis process: (1) The nature of Local Trusted Organisations matters when implementing physical activity leaders programs in the community, (2) Implementation of the JU:MP Leads program, (3) Leadership Development and (4) Importance of relatability in Young Leader development.

### Theme 1: the nature of local trusted organisations matters when implementing physical activity leaders programs in the community

Physical activity/non-physical activity-based organisations were found to have experienced different challenges in their mentorship of aspiring Young Leaders. Physical activity-based organisations were found to be more proficient than non-physical activity-based organisations in developing young people as leaders and moving them into leadership roles. This was due to having the structural capacity to embed young leaders in pre-existing physical activity programs, or by adding bespoke elements to existing provision. This helped support the development of increased physical activity provision for girls. For example, through the program, LTO2 added female cricket sessions to their existing cricket provision.

We have been able to increase our offer for Girls Only sessions. We are able to provide at least one session per week throughout the year due to extra staffing available… Due to increase in demand from young girls wanting to play Cricket, we will be looking to increase our offer to girls in future. (LTO2, Qualitative questionnaire)

Non-physical activity based organisations voiced that they had to establish and deliver new physical activity provisions to facilitate leadership opportunities for young people. They reported that this was difficult to do within the time and capacity they had allocated for the project.

…we don't specialise in any sort of sport…. So this was like a new sort of pilot that we're doing… there was a lot of challenges because if I wanted to set up a session with the young people, I had to be there, present. Now I was only working a few hours on this project… I was attending all the sessions with the young people… I was stretching myself…I can't physically do this. (LTO4 Focus group).

The challenges faced by the non-physical activity organisations were compounded by limitations of the overall delivery structure of the program and in that understandings of each organisation's capacities and opportunities for physical activity delivery were not sufficiently established prior to program commencement. Indeed, frustrations were identified within focus groups with LTOs, in that a “one size fits all” approach was perceived to have been adopted. Yet, such an approach was presented as being at odds with how LTOs would have preferred the program to be managed. Instead, LTO1 explained, there was a need for greater autonomy in delivery approach between organisations which would have supported them in delivering the program in line with their organisational strengths.

we looked at a one size fits all model, and that’s not necessarily the case…[they] could have been looking at what all of our niches were, maybe a bit better spending a bit more time on that, to create something more sustainable… So it needs to be, we need to go back to looking at a holistic approach to things rather than like forcing everybody into one box and you must do this and you've got to conform to these, these criterias… (LTO1 Focus group)

The theme suggests that having a thorough understanding of organisations’ strengths could help support the development of a more sustainable and accessible Young Leaders program when delivered in the community. Also, increased autonomy in delivery approach may provide greater opportunities for organisations to support physical activity leadership.

### Theme 2: implementation of the JU:MP leads program

This theme includes three sub-themes: (1) program scheduling (2) framing and (3) flexibility.

### Sub-theme 2.1: Program scheduling

Overall, the program was found to be well accepted by the Young Leaders and their mentors. We found that an acceptable method of supporting Young Leadership development was to frontload formal training and focused leadership development opportunities by positioning them within the first half of the program delivery period. This process maximised the potential for Young Leaders to undertake more delivery in the latter part of the program, which coincided with summer and the organisations’ busier delivery months. For all organisations, due to their involvement in Holiday Activity and Food (HAF) programs during the summer months, this was a period when they had the most engagement with local children and families.

The main elements will be the sessions because, without the courses at the start of the year, we wouldn't have been able to do any of the sessions [in the summer].. we've been able to get more sessions and get to know more people. (Aakash, Male)

The young people have been delivering four-hour sessions during the summer holidays on Friday’s, Saturday’s and Sunday’s (LTO4 questionnaire).

### Sub-theme 2.2: Framing

The program was well accepted overall. Yet, there was a disconnect found between the LTOs and young people in their understanding of the program's focus; LTOs indicated the program was about delivery and they expected prior competence, whereas young people viewed the program as developmental.

We advertised it as an opportunity to get experience and training but it is paid work so if I was to advertise again I would advertise it as a job vacancy. (LTO3, Qualitative questionnaire)

… everyone has to start somewhere… [and] it's just showing that you know young leaders, yeah, you do tend to fall under a lot, everyone does. But it just shows that young leaders, no matter what happens if you make a mistake, because or no matter you make a mistake, or if you like, do something wrong. That's not effective. Doesn't matter. Just keep going. (Joseph, Male)

Nevertheless, the program supported the development of competent Young Leaders who could support community provision.

Our list of activities that they did, should be able to send them off into a school and that is literally what it has done. So when I go to schools and say, ‘we can run an after school club through the JUMP program, you're in the area, would you be interested?’ I give them the basic qualifications that we've done through this and they're like, ‘Yeah, that's fine’… **(LTO1, Focus group)**

The framing and delivery ethos of the program was found to be integral to its acceptability. The program offered a safe and supportive environment for development and in this respect offered a ’soft entry’ into physical activity leadership.

…My [leaders] are shadowing me [who's been] a coach for 17 years now… Things that I've experienced that I can say ‘I'll watch out for this and think about this’..they're not just being thrown in the deep end. They're having somebody watch their back… **(LTO1, Focus Group)**

…this is a good environment for us to learn, because it's not too demanding, in the sense that if we make mistakes, it's not going to be detrimental **(Nusha, Female)**

Owing to this support, the program served as a community of practice in which Young Leaders felt psychosocially safe and supported.

I think we built a very good relationship as a team…, it's nice knowing that we've done that…It's all just been love and support. Yeah. pushed each other…[we’ve] had each other's back when needed. **(Iram, Female)**

We all are communicating the plan now. ‘How are we going to run the session? Who's gonna do what?’ If anyone needs help, we help each other out… **(Joseph, Male)**

**Figure 2:**
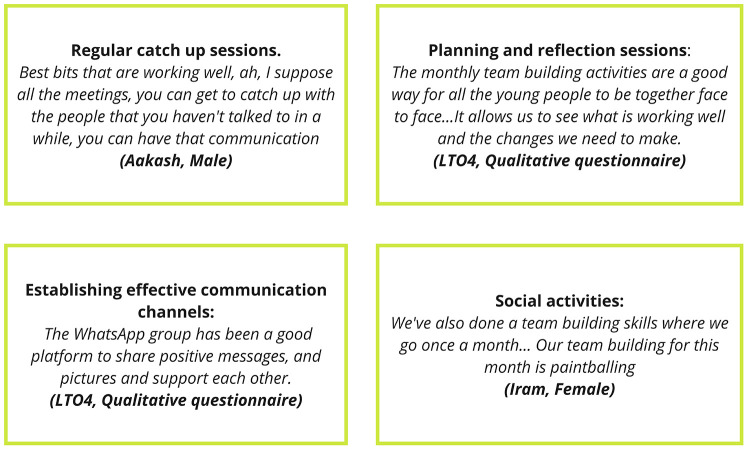
Program components identified in REM.

Figure 2 outlines mechanisms that were found to frame the program as a community of practice.

The mechanisms outlined in Figure 2 were embedded in the program, and were adopted by all organisations. However, in line with our realist stance, it is notable to mention how one LTO took an extra step to support leadership development by structuring their delivery of the program. LTO1 implemented a tiered approach to leadership development, which featured three classifications. This tiered approach was found to be a sound example of a safe and supportive model of supporting transition into independent physical activity leadership whereby a young leader would be capable of supporting physical activity without supervision.

Tier 1 (Observation):

The leaders observe sessions and carry out small tasks during sessions such as setting up drills and taking registers, etc

Tier 2 (Assistant coach):

Leads help lead sessions, they will deliver either warm-up, skill or cool-down.

Tier 3 (Lead Coach):

Leads are confident to deliver by themselves and lead full sessions

(LTO1, Qualitative questionnaire)

These tiers represent the leaders’ level of development, from observing and supporting delivery to assisting physical activity leadership under supervision, through to independent leadership.

### Sub theme 2.3: Flexibility

Within the context of the program we found that the flexibility in delivery was an important aspect that supported engagement and development. Where there was limited flexibility in delivery, managing the program around Young Leaders’ variable schedules and competing priorities was a challenge for LTOs.

The biggest challenge has been organising sessions in line with the young leaders’ other commitments (LTO4, Qualitative questionnaire)

We found that the contractual constraints of a six hour delivery allowance per young leader was rigid and limited the appeal of the program for a wider range of young people. LTO1 considered how the contractual restriction had contributed to one young leader withdrawing from the project.

Only offering six hours of paid work per week this isolates a lot of the young people. We have had one of our really promising leads who has had to give his place to someone younger as he is getting married shortly and needs a bigger income so has now got a full-time job at TESCO. **(LTO1, Qualitative questionnaire)**

The Young Leaders had competing professional and educational commitments which conflicted with fixed program components of the program such as training which were only possible to deliver on specific weekends. However, where program flexibility was evident this supported young leader engagement. Some program components were more acceptable to Young Leaders as a result of their flexibility, including the delivery of physical activity.

When it was GCSE time? so the two weekends that had the multi skills because I had to catch up. I had to just like sacrifice two weekends. So basically [with the rest of the program], with my mum, spoke to (name) she's like is it okay if she does [her delivery] after GCSE because she will be completely free because after GCSE you get three full months, she said yeah that's fine. I'll just give her more [delivery] sessions then instead of now. **(Maya, Female)**

This indicates that when delivering Young Leaders programs within the community it is important to consider how young people will engage in leadership development in line with their aspirations and competing priorities.

### Theme 3: leadership development

**Figure 3:**
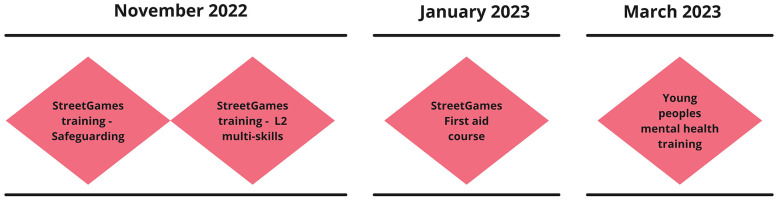
Core training program schedule.

We found that Youth Leadership development aligned with Positive Youth Development, particularly by supporting the development of confidence and competence amongst the young people. The program developed Young Leaders’ formal competencies through a range of nationally recognised qualifications (see Figure 3), which for insurance purposes enabled them to lead physical activity in schools and community settings.

These are courses so that like when you are coaching you have to have safeguarding and first aid so they are a central thing you have to have [to deliver in schools and communities]. (LTO1, Focus group)

Through mentorship, the Young Leaders developed additional, informal competencies including interpersonal skills, behaviour management strategies and an understanding of local physical activity delivery.

Mostly reading [people] after something’s happened… just looking at some people to see how their expression changes, [then thinking] Yep, I need to change the subject slightly but still keep within the topic… It’s an understanding [of] what to do after that, in terms of safeguarding (Ishan, Male)

[it has] improved my communication skills with little children… I don't [usually] work with children… it’s given me, it’s helped me improve my communication skills (Samaira, Female)

The impact of this was carried across to other areas of the young people's lives.

It’s learning to deal with a lot of people in general. So [because of what I've learnt] even in my normal life now I just think well someone might not understand so I might have to do it in a different way. So yeah, a lot of things I've learned from this apprenticeship, not just sport, just in general learning to deal with people. (Maya, Female)

In the initial stages of the program, the leaders lacked confidence generally, and in the early stages of the program, this presented itself through young people struggling with communication. This initially impacted their engagement with the program and their mentors. LTO3 reflected on this during the initial focus group.

I think for me personally, the young people are more challenging than I expected… in terms of communication… they’re not responding to calls. They’re not responding to messages… it’s the biggest challenge right now. (LTO3)

Likewise, some of the young people were described as lacking motivation.

…the [young] people that we’ve got [on the program] they do lack a lot of motivation. (LTO4)

This limited confidence and motivation was interconnected specifically with young people's recognition of their capacity to become physical activity leaders.

*I think if you asked me a couple of years ago, would you be interested in coaching? I would’ve been like, No, I don't think I can do that. Whereas now I am like, you know, I am capable of doing that **(Samaira, Female)***Increased confidence was consistently described and attributed to program elements including sustained professional and pastoral mentorship, opportunities to shadow other coaches, exposure to unfamiliar leadership situations, opportunities to practise leadership in psycho-sociologically safe environments, opportunities to engage in critical and reflective discussions, and an opportunity to work with mentors to develop problem-solving skills.

I've gone through a rough year personally…, so I wasn't too sure [about it] I was quite broken, and I didn't know what I wanted. I was just all over the place. I started this course… I enjoyed it immediately. I thought you know what, I do know what I want. And just delivering and getting excited about sessions coming up. I get happy. I'm not going to lie I get happy planning the session. So I feel like it’s helped me so much with my confidence. And it’s put me in a better place. [I think] you know what, I can do it and I'm gonna do it. (Iram, Female)

Increased overall confidence appears to support young people's perceptions of their leadership capacity. Specifically, elements such as planning and adapting sessions for different audiences, learning how to interact with parents, and being prepared to take on unfamiliar roles was found to greatly improve when leaders developed greater self-confidence and autonomy.

Like I said earlier… there was a point where I wouldn't even like to speak in front of people. Now [I’m] taking the lead in terms of what session you know, session planning, for example… I'm taking leadership in where you know, I kind of manage the game and tell the girls you know, what positions, they need to know how many overs it is. So I think that’s been a massive impact on me [and] a positive impact. (Samaira, Female)

The leaders experienced shifts in their identity and understanding of their positionality within their communities as a result of the program and their development. Indeed, the young people began to understand themselves as leaders and role models and became more comfortable with the responsibility this entailed. In this regard, the young people viewed themselves as being able to ’step up’ and set an example for local young people.

I think this sports leader, its when… your participants look up to you when they can look up to you and they feel comfortable around you, and they will talk to you. and I feel, I feel as a leader (Iram, Female)

The development of competence and confidence, as well as being embedded in local organisations, and holding positions of authority as physical activity leaders were further catalysts that supported these redefinitions.

[The program] made me kind of realise that… I'm 21 years old… I'm not obviously at that level where I feel like I've got so much authority. But when you work with these children and then they say, coach. Yeah, you have got a responsibility over [them]… So it’s changed the way that I kind of act… the least I can give them is professionalism, and I have to step up to that. (Nusha, Female)

Growth in knowledge, and the acquisition of experiential physical activity delivery skills embedded identity change. Yet the identity of being a leader was embedded further through the immersive experience of leading young people in physical activity and in being labelled accordingly.

[Through the program, I realised] I am, you know professional, whereas in university, you don’t get that sense. You’re not going to get [people] calling you sir and coach. (Nusha, Female)

The adoption of a new identity was emphasised by young people such as Iram securing further employment and leadership opportunities aligned with the program.

We have had one who has moved on to work at [local Rugby club] cus he’s got these qualifications… We finished this course [now im a] Directors of [the LTO I worked for]. Oh, yeah. I'm one of the directors. Yeah. And so I am coaching in different places, different primary schools, secondary schools, community centres. SEN departments…Yeah…. Its a full-time job (Iram, Female)

### Theme 4: importance of relatability in youth leadership

Positioning young people as physical activity leaders in the community leveraged their relatability as role models allowing them to make impactful contributions to the physical activity landscape. This approach was seen to improve accessibility of local physical activity provision for local young people by harnessing the relatability of young people as leaders. Yet, it was also viewed as establishing the Young Leaders as positive role models who could have influence within their community:

I have watched these young people become peer role models to each other and step up and embody what a community coach is… They have also become a role model for our participants. We already have a waiting list for the next time this project runs, and other young people from the sessions our Leads have delivered have expressed how they would like to join the program. (LTO1, Qualitative questionnaire)

Relatability was found to give the Young Leaders the unique potential to inspire other local children and young people. Factors such as age and ethnicity formed the basis of the leader's relatability which served as motivation for local children and young people to engage in physical activity and be more likely to see physical activity leadership as a viable career path.

…I think it’s raising more awareness, to be honest, because you've got young people who are doing it…You can get quite a lot of other [young] people involved. You can say, uh, you know, this is what I'm doing… it’s something you can let people know and raise awareness of as well. I personally think I think if you've got people from different backgrounds, and different ages, it’s just raising more awareness and it’s just showing capabilities as well. (Samaira, Female)

I think [young leaders is]… that thing that, [it’s a message like] I can do it, you can do it. I'm able to stand here and do this. And so if you can do it, I just think it’s given them that somebody of their own age somebody or their own time, has been able to achieve this and if you work hard, you do it. You're able to achieve it as well. (Iram, Female)

Relatability was paramount for leaders having a positive impact in the community, and was viewed as a key pull factor within the program, which could support local young people to engage in physical activity. This was explicitly tied to ethnicity as positioning south-asian young people in positions of authority within the local community was seen to be inspirational for other local south-asian children.

Ah, being a brown person, there isn’t much role models like myself in our community… because growing up in my area there was, you see drug dealers everywhere on the streets. Ah, loads of rubbish, just the youth acting up and just causing havoc in the community. So I wanted to help them by giving them a role model that they can follow, that’s doing something with their life. (Aakash, Male)

Em, I think it’s because when I look around in my community there’s mostly British Pakistani people like me or British Indian people like me where and yeah. Em, so if they see ah, a brown person that’s doing something with their life, they think oh, I might be able to do this as well or can do this, I can achieve a certain level… that they've gone through. (Aakash, Male)

Relatability was particularly important for female leaders in the program who had a distinct potential to mobilise their relatability for positive effect. Indeed, there was particular value in allowing South-Asian girls from the local community to see ’someone like them’ in physical activity leadership roles so as to highlight physical activity as a viable career and/or recreation route.

it’s not very common within our with British Pakistani [girl] who wears a hijab to go into sport. So it’s something really good, opens up doors for many girls to be able to do it and gives confidence to many other young people that she can do it. So can you (Iram, Female)

…in our own culture, there’s not a lot of females… that stand in these industries.… Being a British Pakistani wearing the hijab as a Muslim… it gives opens them doors for other girls who just thought you know what? Yeah, we can do it because you don't see that many role models out there So then you see them type of girls coming in… I think it does make a difference. Especially because… you don't see a British Pakistani you won't see a hijab standing on one of the top boards… It’s not plastered everywhere, is it? (Iram, Female)

## Discussion

This research examined the implementation of a Young Leaders program known as JU:MP leads in Bradford, UK. It found that a community-based leadership development model has the potential to empower local young people from deprived communities to develop as physical activity leaders byfostering markers of social capital (10, 21, 40). Moreover, it demonstrates that when a flexible delivery model featuring formal qualifications, mentorship and leadership opportunities is implemented, Young Leaders programs can support positive youth development within deprived communities. This paper provides practice-based evidence that targeted and structured community-based Young Leaders programs have the potential to reduce inequalities, particularly related to the intersection of ethnicity and gender in accessing physical activity leadership, building on the extant literature (5). the Tresent study also aligns with calls to broaden physical activity-based youth employability programs (39, 66). Providing nationally recognised qualifications and other attributes of employability, increased the social mobility of the young people who took part, highlighting theprogram's value (67). However, like previous youth programs, JU:MP Leads struggled to move beyond leadership development into broader civic engagement and systemic change (45). Mechanisms such as evaluation sessions, and the components highlighted in Figure 2 supported such transition yet it was limited. To further embed this, young leaders should be engaged more authentically in the design and delivery of projects like JU:MP lead, taking ownership of their own development. This could be further embedded by supporting young people to mobilise their relatability, ideas and passions to address local political issues, such as South Asian female representation in sports, and the local mores of drugs. Whilst there may be tension in developing young physical activity leaders in deprived and under-resourced communities (25) from the PYD perspective the matter of how to position young people to address such engrained systemic issues may be a more significant tension. Further research is needed to purposefully design interventions to address this.

Within our analysis, we identified a lack of congruence between the organisation's preconceptions of a young leader and the philosophy of the program. All LTOs expected that the aspiring Young Leaders would have a high baseline level of competence at recruitment and that they would be ready to “hit the ground running” at the outset of the program, with their formal training linearly increasing their competency. In reality, many initially lacked self-confidence, and key professional skills like communication and motivation. This initially limited their capacity to engage in the program in the early stages. This frustrated LTOs who expected aspiring leaders to be motivated, enthusiastic, and autonomous; yet this understanding is at odds with PYD. One aspect of PYD is that young people possess an innate capacity to contribute to themselves and society, and that such capacity can be developed through engagement with environmental components including local trusted organisations. In taking a PYD perspective it is vital to recognise personal development and contribution as markers of PYD having occurred (31).

The disconnect between the LTO's and PYD theory highlights the importance of defining the purpose of community-based Young Leaders projects that bridge the gap between increasing local physical activity delivery and providing development opportunities for local young people. These priorities need to be communicated and carefully managed on an ongoing basis. Because as seen in the literature (68) imbalanced expectations in programs featuring partnership working can lead to disappointment, lack of direction, and lack of cohesion in evaluating program efficacy. Our research also emphasizes the importance of establishing clear timescales to ensure effective transitions from leadership development to independent leadership, where young leaders can manage physical activity without direct support.

In JU:MP leads, prioritizing the first half of the program for development and the second half for delivery (with continued development) proved effective. This approach supported PYD by building confidence and competence towards delivery in the first half and supporting local physical activity delivery in the second. The program's Sept-Sept schedule allowed development during winter months, setting up increased physical activity delivery in summer. This seasonal approach supported sustainability withleaders able to deliver on summer programs such as Holiday Activity and Food (HAF) and in accessible and free-to-use venues such as parks. Not all such programs will have this opportunity, yet future youth physical activity leadership programs should consider how best to manage scheduling to create an appropriate balance between development and delivery.

Engaging more disengaged young people and those who lack confidence in Youth Leadership development supports PYD by acknowledging the potential of all young people (31). By supporting underrepresented young people, physical activity leadership development programs can support greater diversity in the Youth Leadership workforce and challenge narratives about such opportunities being best suited for confident and outgoing young people (13). The “three-tier model” implemented by LTO1 within the program was considered effective in supporting a “soft and safe” transition into physical activity leadership for young people. This approach was considered particularly effective as it aligned with PYD in facilitating immediate opportunities for young people to support physical activity leadership yet supported young people to transition into more independent leadership roles. Through this approach, young people could quickly begin to gain experience in leading physical activity. This approach provided a supportive structure on which to harness young people's capacity as leaders in the present (30) and to support them in developing as independent leaders of the future (69, 70). Future youth leadership programs should consider adopting a tiered approach to support this transition.

Promoting PYD principles through the program supported locally rooted young people in developing identities as leaders. The key mechanism supporting this development was the program's capacity to harness the leader's inherent motivations to engage (31). This mechanism was supplemented by factors including nationally recognised qualifications aligned with physical activity leadership and being placed in positions of power in LTOs. These mechanisms enabled the Young Leaders to establish visceral roles as *leaders* that went beyond simple physical activity delivery. They grew to view themselves as role models leading the way for “young people like them” to develop an affinity for physical activity. This is significant as current discourse highlights that relatability between leaders and participants is an important aspect of physical activity enjoyment (7). The potential of this was considered to challenge local mores of crime and drugs and to deconstruct traditional ethnic and gender norms that have positioned physical activity as taboo for certain groups, not least South-Asian girls, the value of this cannot be understated (14, 18). In this respect, placing local young people in leadership positions through physical activity can begin to deconstruct social justice based adversity (27, 30, 38). However, these considerations are idealistic, and targeted interventions are needed to bring about more substantial systemic change.

We found several mechanisms underpinning Youth Leadership development in a community with high levels of deprivation. For example, flexibility and relevance were crucial for the program's impact on young leaders. The 16–25 age group presents logistical challenges for program deliverers. Competing schedules, varied commitments (e.g., work, college, school, and religion), and social challenges (e.g., young people wanting to spend time with their friends on a weekend) make it difficult to deliver a structured training program in the community context. However, through a flexible delivery model 20 young people progressed through JU:MP Leads to develop as competent physical activity leaders. These challenges were outweighed by the value of developing relatable young people who can lead physical activity and serve as role models for local young people. To align with PYDs inclusive ethos, it is essential to deliver flexible “bottom-up” projects that support young people in managing competing priorities. This reinforces the importance of giving young people ownership and autonomy in their leadership development (77).

Though JU:MP leads, combining formal training and ongoing mentorship to create a safe and supportive ethos provides an ideal platform for young people to progressively develop as physical activity leaders. The formal training components gave the leaders a solid platform upon which to pursue physical activity leadership in the short and long term (30). Yet, this research paper highlights that mentorship can be impactful in supporting leaders’ personal development and facilitating their growth into Young Leaders. The mechanisms outlined in Figure 2 provide a sound foundation for future Young Leaders’ mentorship.

This research supports findings from a recent Delphi study, particularly that young physical activity leaders should be relatable to the local communities they are embedded in (13). However, when viewed through the lens of PYD, the data from the present study provides contention to several of the consensus statements outlined in Steward et al. (13). A key point of difference is the notion that young people should possess several pre-requisite traits such as confidence and responsibility at the outset of their engagement, meeting a certain standard to be considered for recruitment. We suggest that a more nuanced understanding is required. In this research, lacking confidence and self-responsibility at the outset of the program was typical of the young people who engaged in the program. Yet throughout, they developed these competencies and took on roles as young physical activity leaders. PYD says that all young people can develop into leaders when embedded in supportive environments and given the resources to maximise their potential (14, 31) and JU:MP leads stands testament to that. Rigid recruitment standards contradict this approach. Thus, future programs should (i) recruit young people based on their enthusiasm to engage, and their alignment with the rationale of the program, and (ii) be more explicit with their assertion of the skills young people will develop through their engagement. We believe that this will make physical activity leadership more accessible to underserved groups, and harness the untapped potential of young people to support local physical activity delivery (71).

Despite the recognised value of empowering young people by developing them as physical activity leaders, a persistent gap in sustainable funding remains. This reality undermines the potential for such initiatives to be scaled and have longer-term impact. As with JU:MP leads, political support in the way of funding often materialises around strategic intention with programs often designed for deficit reduction purposes such as tackling inequalities, instead of asset-based approaches such as broader and meaningful youth empowerment. This disconnect results in short-term programs that are limited in their potential for impact with young people having limited support and resources to realise wider systemic change. For real progress, sustained investment in youth leadership development is essential, enabling succession planning and supporting young people to become embedded catalysts of change within the wider physical activity workforce. Further governmental funding is necessary to demonstrate a genuine commitment to youth empowerment.

### Strengths and limitations

The use of qualitative methods in this research is a particular strength. By implementing qualitative methods we were able to reveal the social and cultural contexts that influence participants’ and providers’ experiences of the program. The methods and participant groups involved in this research shaped a holistic understanding of the program. Through interviews with young people we developed an understanding of their engagement in JU:MP leads. By engaging LTOs we were able to explore the implementation of the program, and by implementing REM, we were able to bring young people, LTOs and the program delivery partner together to explore contribution/attribution relationships within the program which supported our realist evaluation approach. A key limitation of this research is the omission of follow up data collection to understand any potential sustained impact of the program. Furthermore, the findings from this research may lack generalisability due to the JU:MP leads program involving only British South Asian young people and because the research is focused on one locality.

### Recommendations

Our recommendations for practice are to:

1. Take an inclusive, PYD-rooted outlook to participation and work with local young people who are representative of the local contexts and motivated to engage and develop.
2. Explicitly outline priorities at the outset of the program to maintain a relevant balance between development and delivery. A strong approach would be to prioritise development in the first half of a program and delivery in the second.
3. Deliver a core training package that readies young people for delivery alongside mentoring that supports them in taking on leadership roles.
4. Utilise a tiered approach to facilitate leadership that acknowledges individual young people's training needs and progressively supports them into leadership roles.
5. Ensure flexibility within program delivery to meet young people's needs.

Future qualitative research should be undertaken to explore post-program engagement to explore the longer-term impacts of Young Leaders’ projects. Moreover, future research should be undertaken to tailor youth physical activity leadership programs for underserved groups, such as South-Asian and eastern european girls, and non-binary and gender minority young people. Lastly, qualitative research could explore community perspectives on Youth Leadership development programs to understand how such programs may impact communities, and how they can be developed to maximise impact.

## Conclusion

This research meets academic calls for in-depth exploration of community-based youth physical activity leadership programs (13). Using Interviews, focus groups and REM we evaluated a community based young physical activity leaders development program delivered in Bradford, UK. The program, known as “JU:MP leads” developed 20 young people aged 16–25 as Young Leaders between September 2022 and September 2023. This research demonstrates that a community-based Young Leaders development program can be effective in supporting local young people from deprived communities to develop as physical activity leaders. Key mechanisms within the program that supported development included the acquisition of formal, nationally recognised qualifications, informal training and mentorship, peer support and ongoing reflection. Moreover, relating to program mechanisms, our findings highlight the importance of having a flexible program design in which young people can engage around other commitments.

## Data Availability

The raw data supporting the conclusions of this article will be made available by the authors, without undue reservation.
